# IgE Reactivity to Common Cypress (*C. Sempervirens*) Pollen Extracts: Evidence for Novel Allergens

**DOI:** 10.1097/WOX.0b013e3181eb3525

**Published:** 2010-08-15

**Authors:** Youcef Shahali, Jean-Pierre Sutra, Gabriel Peltre, Denis Charpin, Hélène Sénéchal, Pascal Poncet

**Affiliations:** 1 ESPCI ParisTech, PECSA, UMR CNRS 7195, Paris, France; 2 Hôpital Nord, Marseille, France; 3 Inserm, Paris, France; 4 Pasteur Institute, Paris, France

**Keywords:** *C. sempervirens*, cypress pollen, water insoluble allergens, immunoblotting patterns

## Abstract

**Background:**

Cypress pollen is becoming an increasing cause of respiratory allergy in some regions worldwide.

**Objective:**

The aim of this study was to determine some of the main allergens implicated in the common cypress (*C. sempervirens*) pollen allergy.

**Methods:**

Pollen extracts were optimized by using some detergents and chaotropes in order to solubilize both water and non-water soluble proteins. *C. sempervirens *pollen extracts were resolved by one and two dimensional electrophoresis and assayed with sera of allergic subjects.

**Results:**

Five predominant allergens with apparent molecular masses ranging from 14 to 94 kDa were detected. Two principal IgE-binding patterns were clearly distinguishable: a first one represents patients with a heterogeneous IgE reactivity to several allergens (pI 3.5-8.5) with molecular masses ranging from 35 to 94 kDa (HMW). The second one corresponds to little less than 50 percent of tested patients with specific IgE binding to 2-3 spots (pI 10-11) of about 14 kDa and weak or no reactivity to HMW allergens.

**Conclusion:**

The extraction of water insoluble proteins allows the revelation of novel allergens as well as different allergen sensitization patterns in the *C. sempervirens *pollen allergy. These novel IgE reactive components may subsequently be applied to expand the panel of well-defined cypress pollen molecules for a more efficient allergen-based diagnosis and therapy.

## Introduction

Cypress is the general name given to many plants belonging to Cupressaceae (cypress family), the most wide-spread of all gymnosperm families. Because of their exceptional potential of adaptation to various climatic and edaphic conditions, cypress trees and shrubs are distributed in diverse habitats on all continents around the world.

Allergy to cypress pollen was put forward for the first time by Black in 1929, [1] who demonstrated the role of mountain cedar (*Juniperus sabinoides*) pollen in the induction of hay fever in Texas and southern states of North America. Since then, cypress-cedar pollen allergy has been reported in numerous countries located in various geographical areas such as South Africa, [2] Australia, [3] France, [4] Italy, [5] Spain, [6] Marrocco, [7] Israel, [8] Albania, [9] Greece, [10] Turkey, [11] Iran, [12] and Japan [13] and is currently known as an increasing cause of pollinosis worldwide [14].

However, the underestimation of the real prevalence of cypress allergy is still a matter of concern. This fact could be partly explained by 1) the lack of satisfactory diagnostic extracts, [15,16] 2) the influence of environmental and anthropogenic factors on pollen allergenic properties, [17-19] 3) the overlapping of symptoms with those induced by common winter diseases, [20] and 4) the presence in the atmosphere of submicronic vectors of allergens originated from the cypress pollen sac called orbicules. These particles are found all around the tapetum (the nutritive layer composing the inner wall of the pollen sac) and they are in close contact with cypress pollen grains [12,21]. Because of their small sizes (300-600 nm) the concentration and persistence of these spherical fine particles during and after the pollination period is difficult to estimate.

## Common cypress pollen allergy

In Mediterranean countries, the common cypress (*C. sempervirens, Cup.s*.) also known as Italian or funeral cypress constitutes large natural forests in oriental Mediterranean areas (eg, Greece, Turkey, and Albania). It also plays a central role in the rural economy. For instance, it is widely cultivated as wind or erosion barrier (protecting efficiently many other plantations), source of timber, and functional hedges. In urban areas, *Cup.s*. trees are often present as ornamental in both private and public green spaces. Cypress pollen is currently estimated to represent 20 - 40% of the annual pollen atmospheric concentration in regions surrounding the Mediterranean Basin [9,22].

Although the characterization and standardization of *Cup.s*. pollen extracts have been the subject of several studies, up to now, only 2 *Cup.s*. allergens have been well characterized:

1. Cup s 1, a 45 kDa protein among the pectate-lyase family, currently recognized as the major allergen of *Cup.s*. pollen [23]. This protein shows a high degree of sequence homology [24] with major allergens of other cypress allergenic species (ie, Cup a 1, Jun o1, Jun v 1, Jun a 1, Cha o 1, Cry j 1).

2. Cup s 3, a 34 kDa thaumatin-like protein, [25] reported as pathogenesis-related (PR-5) revealing a high cDNA homology (95%) with Cup a 3, a protein showing increased expression under polluted air conditions [17,19].

## Objective

In the present investigation, we aimed to determine some of the main allergens implicated in the common cypress pollen allergy. The extraction methods currently used in the preparation of cypress pollen extracts fail to give access to hydrophobic proteins. To bring to light the potential role of most insoluble fractions in the cypress pollen allergy, we choose a proteomic approach based on the extraction and solubilization of both hydrophilic and hydrophobic proteins by using some chaotropes and detergents.

## Materials and methods

### Patient's Sera

Sera of cypress allergic patients were selected according to their symptoms, positive skin prick test results and positive ImmunoCAP (Phadia, Upssala, Sweden) with serum-specific IgE ≥ 0.71 kIU/l (CAP class ≥ 2). Sera were partly collected from biologic analysis laboratories and represented residues of IgE titer evaluations. Another part was drawn, after obtaining an informed consent, from individuals suffering from cypress pollen allergy in the south of France (Nord Hospital, Marseille). For each analysis, the serum from a healthy individual with a normal total immunoglobulin E (IgE) concentration (≤ 0.35 kIU/l) has been selected as control.

### Cypress Pollen Extracts and Protein Studies

*C. sempervirens *pollen was supplied by Allergon AB (Angelholm, Sweden). For the preparation of pollen extracts, 100 mg (1/10 wt/vol) were incubated overnight and under rotation in 1 mL of the solution I (38 mmol/L^-1 ^Tris pH 6.8 containing 4% (wt/vol) SDS). The suspensions were centrifuged at 14,000 *g *for 20 minutes at 4°C and the supernatants were collected and stored as aliquots at -20°C until use. To remove SDS for isoelectric focusing (IEF) separations and other interfering substances present in pollen extracts (eg, salts, carbohydrates, and phenols), all samples were treated with 2D Clean-Up kit (GE Healthcare Bio-Sciences AB, Uppsala, Sweden) according to the manufacturer's instructions. The resultant dry pellets were resuspended during 1 hour under rotation into either the solution I for one dimensional electrophoresis (1-DE) or, for subsequent IEF, in solution II: 7 mol/L^-1 ^urea, 2 mol/L^-1 ^thiourea, and 2% (wt/vol) of 3-(3-cholamidopropyl)dimethyl ammonio)-1-propanesulfonate (CHAPS). Protein concentration of samples was measured by the Bradford protein assay [26] using bovine serum albumin (BSA) as standard.

### One-Dimensional Electrophoresis (SDS-PAGE)

Extracted proteins were applied to a thin 8-18% gradient polyacrylamide gel (ExcelGel, GE Healthcare, Uppsala, Sweden) and run on a flat-bed electrophoretic chamber (Multiphor II, GE Healthcare Bio-Sciences AB, Uppsala, Sweden) at 15°C. The gel was partly transferred to a nitrocellulose membrane for Western blotting assays and another part was stained by Coomassie blue for detecting the separated proteins. Molecular mass markers ranging from 14.4 to 94 kDa were used as comparative references.

### Two-Dimensional Gel Electrophoresis

*Cup.s*. pollen extract was first separated by IEF performed in a polyacrylamide gel 4%T, 3%C (Clean gel, GE Healthcare, Uppsala, Sweden) hydrated in the solution II containing 5% vol/vol Servalyt pH 2-11 (Serva, Heidelberg, Germany) on a flat bed electrophoretic chamber (Multiphor II) cooled at 15°C.

Five-millimeter wide strips of the focused gel were cut, incubated in the equilibration buffer (114 mmol/L^-1 ^Tris pH 6.8 containing 12% (wt/vol) SDS) and submitted to the second dimension: a SDS-PAGE separation on an 8-18% gradient gel, allowing a wide-range separation according to molecular masses. Two-DE gels were either silver-stained according to Blum et al, [27] Coomassie blue-stained or blotted onto a cyanogen-bromide activated nitrocellulose (NCa) sheet [28].

### Western Blot

Electroblotting of separated proteins was performed onto NCa sheets with a semidry Novablot apparatus (LKB, Uppsala, Sweden) after the manufacturer's instructions (1 hour, 1 mA/cm2). The membranes were then dried and blocked with phosphate-buffered saline (PBS) pH 7.4 containing 0.3% (vol/vol) Tween 20 (PBS-Tw) for 1 hour at ambient temperature. For 1-DE screening, each NCa was then cut in 2.5 mm wide strips that were individually incubated with 1:10 diluted patient sera overnight at 20°C. For 2-DE analysis, the whole NCa membranes were incubated with individual sera (1:10 dilution in PBS-Tw, overnight at 20°C). Each membrane was washed 3 times for 10 minutes in PBS-Tw 0.1% (vol/vol) and incubated with 1:700 dilution of alkaline phosphatase (AP)-conjugated goat antihuman IgE during 2 hours at 20°C (Sigma Biochemicals, St. Louis, MO). The AP activity was detected using 5-Bromo-4-Chloro-3-Indolyl Phosphate (BCIP) and Nitro Blue Tetrazolium (NBT, Sigma Biochemicals) in 0.1 mol/L^-1 ^Tris acetate buffer pH 9.5.

## Results

### 1-DE (SDS-PAGE) Profile of *Cup.s*. Pollen Allergens

**Figure 1:**
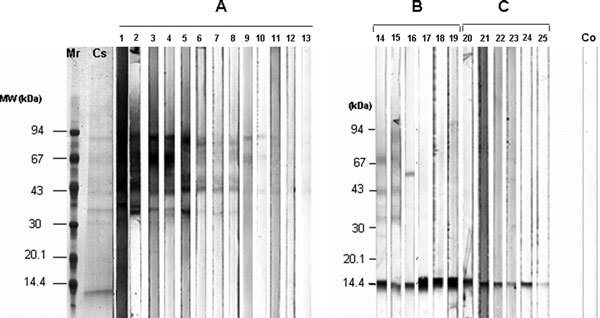
**IgE reactivity to *Cup.s*. SDS-pollen extract**. M represents the marker; Cs, *C. sempervirens *pollen extract; lanes 1-24, screening of 24 patient's sera by Western blotting for specific IgE antibodies to *Cup.s*. pollen extracts and Co, the negative control using the serum of a healthy individual. Strips were ordered by the intensity and frequency of allergen recognition. Differential sensitization patterns were classified in 3 groups: A, represents patients showing heterogeneous IgE reactivity to HMW; B, an intermediate pattern recognizing both HMW and 14 kDa allergens; and C, patient's sera showing a specific IgE reactivity to the protein at about 14 kDa.

The yield of *Cup.s*. pollen SDS extraction was about 1.5 mg protein per gram of pollen, about 17-fold the amount obtained with an aqueous extraction (90 *μ*g/g) [29]. Coomassie blue staining of the SDS-PAGE electrophoresis revealed a wide diversity of proteins ranging from 10 to 94 kDa (Figure 1, lane Cs). Allergen electrophoretic profiles of 24 patient sera with representative IgE reactivity patterns were illustrated in the Figure 1 (lanes 1-24). Recorded data of each patient, including sex, age, ImmunoCAP value, and class along with their other sensitizations to inhalant allergens are given in Table 1.

**Table 1:** Recorded Data of Each Patient

			CAP		
				
Patients n°	Age	Sex	Class	Value	Other Sensitizations to Inhalant Allergens
1	19	F	4	26.2	Grass pollens
*2	11	M	4	31.6	Mites, grass pollen, plane pollen, Alternaria
3	26	M	4	41.1	--
4	14	M	3	12.5	--
5	29	F	3	7.94	Alternaria, tree pollens (oak, linden, chestnut)
6	NA	M	4	21.2	Grass pollens, mites (DF)
7	NA	M	4	18	Grass pollens, mites, mountain juniper pollen
8	NA	F	3	11.5	Grass pollens, tree pollens (olive, cottonwood)
9	NA	M	3	13.9	Grass pollens, mites, olive pollen
10	65	M	3	4.03	Mountain juniper pollen
11	44	F	2	2.87	--
12	31	F	3	3.53	--
13	16	F	3	14.8	Japanese cedar pollen
14	70	M	NA	NA	Cat dander, olive pollen
15	31	M	3	16.5	--
16	19	M	NA	NA	Grass pollens, mites, olive pollen, cat dander
17	13	M	NA	NA	Alternaria
*18	67	M	NA	NA	Grass pollens
19	18	M	3	15.5	--
*20	54	M	3	9.5	Grass pollens, olive pollen, Parietaria pollen
21	11	F	NA	NA	Mites, Alternaria, olive pollen
22	39	F	3	7.96	Grass pollens
23	47	F	3	10.1	Tree pollens (mountain juniper, plane)
24	17	F	NA	NA	Cat dander, olive pollen
25	56	M	NA	NA	--
		10 F, 15 M			18 poly, 7 mono

F = Female; M = male; DF = *Dermatophagoides farinae*; poly = polysensitized; mono = monosensitized; NA = not available. IgE binding to the ImmunoCAP t23 (*C. sempervirens *allergens) are expressed in kIU/l and specific IgE values > 0.35 kIU/l were regarded as positive. Other sensitizations were ordered according to specific IgE levels to different inhalant allergens. *Sera used for 2D-blotting in Figure 3.

**Figure 2:**
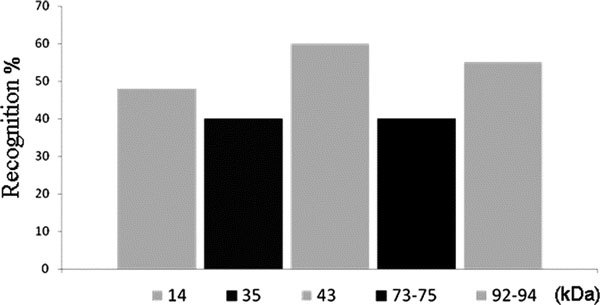
**Histogram representing the frequency of specific IgE recognition directed to different *Cup.s*. pollen proteins in 24 allergic patients**. 1-DE immunoblotting using *Cup.s*. pollen extracts distinguished 5 predominant allergens with apparent molecular masses of 14, 35, 43, 73-75, and 92-94 kDa.

As shown in the Figure 2, 2 main IgE binding patterns were clearly distinguishable. The first one (Figure 1, lanes 1-13) represents patients showing a heterogeneous IgE reactivity to several allergens in the 35-94 kDa mass range [High Molecular Weight (HMW) proteins]. The second one (Figure 1, lanes 14-24) corresponding to little less than 50% of tested patients, reveals a specific IgE binding to a protein of about 14 kDa and a weak or no reactivity to HMW.

Five predominant allergens with apparent molecular masses of 14, 35, 43, 73-75, and 92-94 kDa are clearly visible. The frequency of recognition by specific IgE of allergic patients (*n *= 24) was 60 and 55% for proteins of 43 and 90 kDa, respectively, 48% for the band at 14 kDa and 40% for those of 35 and 73-75 kDa (Figure 2). More than 65% of tested patients recognized one or several HMW allergens (Figure 1, patterns A and B) whereas 48% showed a well-marked reactivity to the 14 kDa allergen (Figure 1, pattern C).

### 2-DE Immunoblotting: Differential IgE Binding Patterns in *Cup.s*. Pollen Allergy

**Figure 3:**
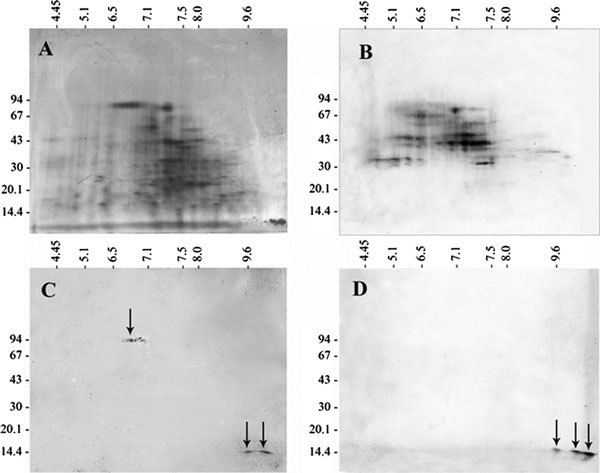
**Silver staining of 2-DE separation (A) and typical 2-DE IgE-binding spectra of *Cup.s*. pollen proteins (B-D)**. First dimension (IEF) was carried out in a 2-11 pH gradient and the second dimension in an 8-18% acrylamide gradient gel. Molecular masses are expressed in kilodaltons (kDa); pI corresponds to isoelectric points. Representative IgE binding patterns B, C, and D correspond, respectively, to 2-DE immunoblotting allergen profiles of the patients 2, 18, and 20 of Figure 1 and Table 1. The pattern 3B is constituted of numerous IgE-reactive proteins with molecular masses ranging from 35 to 94 kDa and pI values distributed from 3.5 to 8.5. The patterns 3C and 3D revealed a very poor heterogeneity (IgE binding protein spots are shown by black arrows) and particularly recognized very basic spots of ~14 kDa.

The 2-DE of *Cup.s*. pollen extracts results in the separation of more than 100 protein spots distributed in a wide range of molecular masses and isoelectric points (Figure 3A). The different 2-DE separation gels were individually immunoprobed with representative sera of different IgE binding patterns (sera number 2, 18, and 20 in the Figure 1 and Table 1 representing the patterns A, B, and C, respectively). As depicted in the Figure 3, the results of the analysis confirmed those previously obtained in 1-DE immunoblotting and led to the detection of 3 distinct IgE-binding patterns to *Cup.s*. pollen proteins: 1) a heterogeneous immunoreactivity to numerous acidic and neutral spots (pI 3.5-8.5) with molecular masses ranging from 35 to 94 kDa (HMW), 2) a specific IgE-binding activity to very basic spots (pI 10-11) with an approximate molecular mass of 14 kDa, and 3) an intermediate pattern representing patients with a well-marked IgE reactivity against the 14 kDa protein and recognizing weakly some HMW. Close series of spots presumably correspond to various isoforms of the same protein.

## Discussion

Cypress trees have a wide geographical distribution and influence the pollen maps of many of the cities. In Mediterranean countries, the cypress pollen is becoming the major source of winter respiratory allergy, commonly inducing symptoms of hay fever, rhino-conjunctivitis, hacking cough, and asthma in sensitized individuals [30]. In Marseille, southern France, sensitization prevalence values of the cypress pollen allergy showed a dramatic increase from nil in 1960 to 25% in 1991 [31]. However, the previous underestimation of the cypress pollinosis because of the low efficiency of cypress pollen extracts commercially available should also be taken into account.

Although several pollen allergenic components have been already described in different cypress species, only 2 *Cup.s*. allergens have been exhaustively characterized and identified [23-25]. This fact could be explained by the very low protein content of *Cup.s*. aqueous pollen extracts making the isolation and characterization of pollen allergens difficult [29].

Until now, most of the reported investigations on pollen molecular allergens were based on the extraction and characterization of water soluble fractions and very few attempts have been made to evaluate the allergenic potency of water insoluble pollen constituents. This trend was mainly established on these 2 principles: 1) when the pollen comes into contact with mucosa of allergic patients, only soluble components are presumed to have the ability to pass through different pollen wall layers allowing their absorption by the respiratory mucosa before the pollen is removed or swallowed; and 2) allergen extracts must be water soluble to be safely applied in the diagnostic and therapy for the allergy without any potential complication that could be associated with some detergents.

However, during the past few years, several water insoluble allergens have been described in some inhalant sources such as *Dactylis glomerata *pollen, [32] gliadins in baker's occupational asthma to the wheat flour, [33] and Hev b 1 and Hev b 3, the major latex allergens located in rubber particles [34].

With regard to cypress pollens, because of the very low resistance of their exine (outer layer of the pollen wall), they are particularly liable to desiccation during their transport, facilitating the exudation and accessibility of both hydrophobic and hydrophilic intrapollinic materials to the environment [12].

Our results show that the use of detergents and chaotropic agents to solubilize insoluble fractions considerably increases the quantity and diversity of extracted cypress pollen proteins and allows the detection of several yet-undescribed allergens. Besides Cup s 1 and Cup s 3, identified on the basis of their expected molecular masses of 43 and 34 kDa, respectively, proteins of 94 and 75 kDa were shown to be IgE reactive and to exhibit numerous isoforms, as revealed in 2-DE immunoblotting (Figure 3B).

A 14 kDa protein was also shown to induce a strong IgE immunoreactivity in 48% of *Cup.s*. allergic patients. Interestingly, this allergen was not detected in patients with high reactivity against HMW allergens. Therefore, 2 patterns of sensitization can be clearly distinguished: a first one represents patients with a heterogeneous IgE reactivity to several allergens (pI 3.5-8.5) with molecular masses ranging from 35 to 94 kDa (HMW). The second one corresponds to little less than 50% of tested patients with a specific IgE binding to 2-3 spots (pI 10-11) of about 14 kDa and a weak or no reactivity to HMW allergens. No obvious relationship appeared between the sensitization patterns of selected patients and their sex, age, symptoms, and poly- or monosensitization.

The very basic isoelectric point of the 14 kDa protein isoforms (Figure 3C and 3D) suggests that this novel cypress pollen allergen contains high amounts of basic amino acids. The nature of this protein and its integrity (an intact protein or a fragment of a larger one) have yet to be investigated; however, the specific IgE reactivity to this protein band allowed the discrimination between 2 distinct categories of patients. Purification and mass spectrometry experiments are currently planned to unravel its nature and will allow the subsequent determination of its clinical relevance by complementary in vivo and in vitro analysis.

In conclusion, water insoluble fractions of *Cup.s*. pollen contains proteins able to sensitize individuals. The molecular mechanism implicated in the immune response to these allergens from their uptake by the respiratory mucosa to their interaction with the cells of the immune system has yet to be clarified. These *Cup.s*. pollen components, newly identified as IgE reactive, may subsequently be applied to expand the panel of well-defined cypress pollen molecules for a more efficient allergen-based diagnosis and therapy.
